# Chilaiditi Syndrome with Carcinoma Rectum: Rare Entity

**DOI:** 10.4103/1319-3767.74443

**Published:** 2011

**Authors:** Vipul D. Yagnik

**Affiliations:** Ronak Endo-Laparoscopy and General Surgical Hospital, Patan -384265, Gujarat, India. E-mail: vipul.yagnik@gmail.com

**Figure 1:**
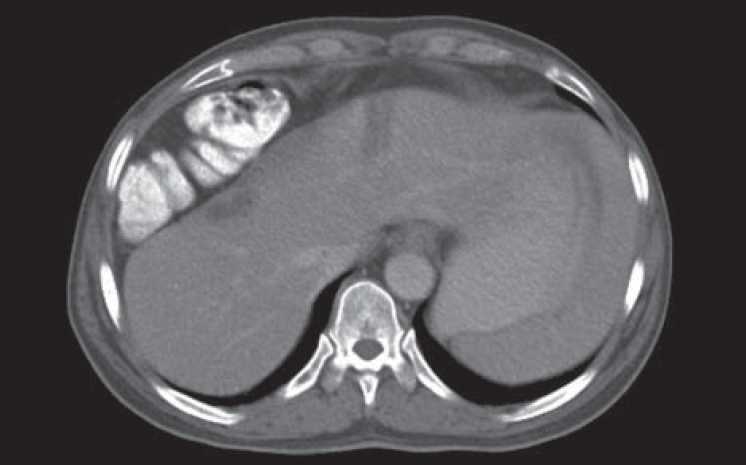
Interposition of colonic loop between diaphragm and liver: Chilaiditi sign

**Figure 2:**
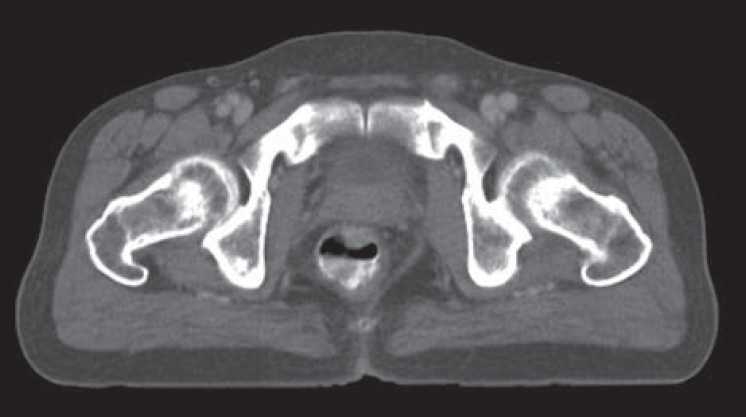
Carcinoma rectum

Sir,

A 70-year-old male presented with complaints of bleeding per rectum since last 6 month. The patient complained of abdominal pain and nausea. He had a history of altered bowel habit and tenesmus. The patient was hemodynemically stable and afebrile. Physical examination showed no sign of peritonitis. Routine blood chemistry was normal. Per-rectal examination revealed circumferential growth of approximately 5 cm from the anal verge. Histopathological examination was suggestive of adenocarcinoma. The patient was subjected to CT scan abdomen which revealed the contrast enhancing lesion in the rectum with hepatodiaphragmatic interposition of the colonic loop [Figures 1 and 2]. The patient was diagnosed to have a chilaiditi syndrome with carcinoma of rectum.

Transposition of loop of large intestines in between diaphragm and liver surface on plain X-ray chest or abdomen is known as chilaiditi sign. Physical examination is usually normal except interposition of loop in chilaiditi sign. Incidence is around 0.1-1%. Symptoms such as nausea, abdominal pain,[1] vomiting, distension of abdomen, shortness of breath in patients with this sign are termed as chilaiditi syndrome. Literature review revealed approximately 160 cases of chilaiditi syndrome. It was first described by Greek radiologist Demetrius Chilaiditi in 1910.[2] Chilaiditi sign may be seen in cirrhosis and COPD. Long redundant mobile colon due to laxity of suspensory ligament of colon or liver is thought to be a contributory cause of chilaiditi sign. Volvulus of the transverse colon[3] or few malignancies (colonic, gastric, pulmonary malignancy),[4] may be associated with chilaiditi syndrome. Subphrenic abscess may show similar characteristic as chilaiditi sign or syndrome. Diagnosis is usually made by X-ray; CT scan will help in confirmation of diagnosis in case of doubt. Management is essentially medical, surgery is offered to those who fail to respond to a medical line of management. The presence of carcinoma of rectum with chilaiditi syndrome requires treatment of malignancy.
